# Nephrocutaneous Fistula Associated With Long-Term Ureteral Stenting and Renal Stones Requiring Nephrectomy With Partial Inferior Vena Cava Resection: A Case Report

**DOI:** 10.7759/cureus.110167

**Published:** 2026-06-03

**Authors:** Masato Yanagi, Yoshihiro Nishikawa, Kuniaki Tanabe, Shuichi Osawa, Jun Akatsuka

**Affiliations:** 1 Urology, Heisei-Tateishi Hospital, Tokyo, JPN; 2 Urology, Nippon Medical School Hospital, Tokyo, JPN

**Keywords:** nephrectomy, nephrocutaneous fistula, pyelonephritis, renal stone, ureteral stent

## Abstract

Nephrocutaneous fistula (NCF) is a rare condition typically associated with chronic infections, such as renal calculi or xanthogranulomatous pyelonephritis. Here, we report a case of NCF in which open nephrectomy required partial resection of the inferior vena cava (IVC) due to severe hilar adhesions. A 64-year-old woman in a prolonged bedridden state following intracerebral hemorrhage presented with fever, pain in the right flank, and purulent discharge from the right lumbar region. She had a long-term indwelling ureteral stent for the right ureteral stones. Contrast-enhanced computed tomography (CT) revealed a renal stone protruding beyond the renal parenchyma with a fistulous tract extending to the skin, consistent with NCF. Marked perihilar fat stranding and hilar lymphadenopathy were also observed. After infection control with meropenem hydrate, an open right nephrectomy was performed. Severe adhesions were observed around the renal hilum. Intraoperative injury to the right renal vein occurred during dissection, necessitating partial IVC resection for vascular control. NCF should be considered in patients with a long-standing infection, renal stones, or prolonged ureteral stenting who present with cutaneous abscesses or persistent flank discharge. Preoperative CT findings, such as marked perihilar fat stranding and hilar lymphadenopathy, may predict severe adhesions around renal vessels. Surgeons should keep these possible complications in mind and be prepared for complex vascular procedures, including possible IVC resection.

## Introduction

Nephrocutaneous fistula (NCF) is a rare clinical entity characterized by a fistulous communication between the kidney and the skin, most commonly presenting as persistent purulent discharge from the flank [1,2]. In general, a fistula forms when chronic inflammation or infection causes progressive tissue destruction, resulting in the creation of a pathological tract between two epithelialized surfaces or spaces. NCF is typically associated with chronic inflammatory conditions, such as renal calculi, xanthogranulomatous pyelonephritis, and renal tuberculosis [1-3]. Chronic infection leads to progressive parenchymal destruction and extension of inflammation into surrounding tissues, eventually resulting in cutaneous fistula formation. Although the kidney is normally separated from the skin by thick muscles, fascia, and retroperitoneal tissues, inflammation may spread through anatomically vulnerable regions such as the lumbar triangle, where tissue resistance is relatively weak. As a result, a fistulous tract can develop from the kidney to the flank skin.

The affected kidney is nonfunctioning in most cases, and nephrectomy is generally the preferred treatment [1,2]. However, surgical management is technically demanding owing to severe inflammation and fibrosis, particularly around the renal hilum.

We report a rare case of NCF arising in the context of long-term ureteral stenting and renal stones in which partial resection of the inferior vena cava (IVC) was required during open nephrectomy due to dense perihilar adhesions. The role of preoperative imaging in predicting surgical difficulty is also discussed.

## Case presentation

A 64-year-old woman with a body mass index of 12.2 kg/m² presented with fever and purulent discharge from her flank area, with flank pain, and was admitted to our hospital. She had been completely bedridden for seven years because of the sequelae of cerebral hemorrhage. She also had a history of hypertension but no history of diabetes mellitus. She had undergone ureteral stent placement four years prior for calculous pyelonephritis secondary to a right ureteral stone, at which time a right renal stone was also identified. The right ureteral stent was replaced every three months. Definitive stone treatment with ureteroscopic lithotripsy was not performed because the patient was completely bedridden with severe extremity contractures, making the lithotomy position difficult to achieve. In addition, she was considered at high risk for postoperative urinary tract infection because of her history of calculous pyelonephritis, severe malnutrition, and poor general condition. Therefore, conservative management with periodic ureteral stent exchange was selected. The abscess was discharged from the skin of the flank (Figure 1).

**Figure 1:**
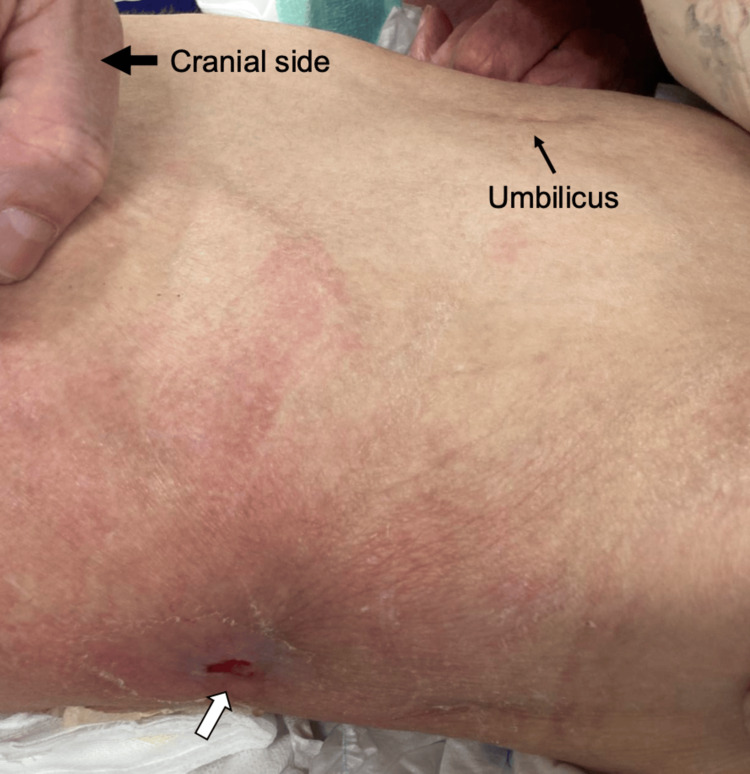
Findings of the skin of the NCF The white arrow indicates a fistula with abscess discharge. NCF: Nephrocutaneous fistula

Computed tomography (CT) revealed a renal stone protruding beyond the lower pole of the right kidney, with a continuous fistulous tract to the skin (Figure 2).

**Figure 2:**
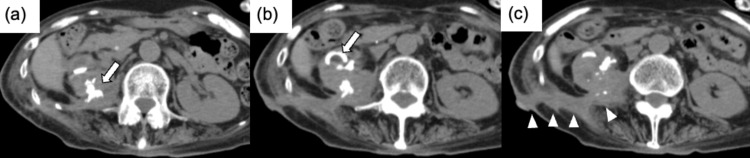
Findings of preoperative abdominal CT (a) The white arrow indicates renal stones. The renal parenchyma is atrophic. (b) The white arrow indicates the ureteral stent. (c) White arrowheads denote the NCF. NCF: Nephrocutaneous fistula

These findings led to the diagnosis of NCF. The ureteral stone also remained present on CT. The renal parenchyma was thinned, and the right upper urinary tract failed to opacify in the excretory phase of contrast-enhanced CT, consistent with a nonfunctioning kidney. Renal scintigraphy, such as MAG3 or DMSA, was considered; however, it was not performed because the findings were not expected to alter the treatment strategy, and the patient’s family declined further evaluation. In addition, CT demonstrated marked perihilar fat stranding and hilar lymphadenopathy with a long-axis diameter of approximately 5 cm (Figures 3a, 3b).

**Figure 3:**
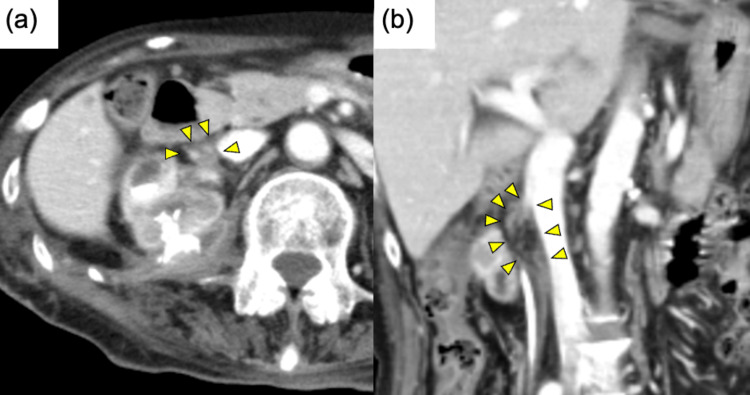
Findings of peri-right renal vein on preoperative abdominal contrast-enhanced CT (a) Yellow arrowheads indicate severe stranding around the right renal vein. (b) Yellow arrowheads depict enlarged lymph nodes in the renal hilus.

Culture of the abscess and urine grew *Providencia rettgeri*. Meropenem hydrate administration was initiated, and four days later, the abscess discharge from the fistula resolved, along with pain and fever. Subsequently, the patient underwent open nephrectomy to prevent infection recurrence.

The patient had been under conservative treatment for a left humerus fracture one month previously and had contractures of the left arm and both lower extremities, making a semi-lateral or lateral position impossible. In addition, strong perirenal adhesions were predicted. Therefore, open transperitoneal nephrectomy was performed through a chevron incision with the patient in the supine position. Intraoperatively, dense adhesions were observed around the kidney and right renal vein. Because of the difficulty in dissection around the renal vein and bleeding due to an inadvertent injury to the right renal vein, the IVC at the right renal vein bifurcation was clamped with Satinsky forceps. The right renal vein side of the IVC was transected, and the IVC was repaired using continuous sutures. Because severe periureteral adhesions made ureteral dissection extremely difficult, most of the ureter, including the persistent ureteral stone, was left in situ. In addition, the adrenal gland was preserved. After kidney removal, the stones in the fistula were removed, necrotic tissue was debrided, and the operative field was then carefully washed with 5000 mL of saline. The operating time was 169 min, and the estimated blood loss was 955 mL. Gross examination of the resected specimen revealed a fistula on the dorsal side of the renal specimen filled with stones (Figure 4).

**Figure 4:**
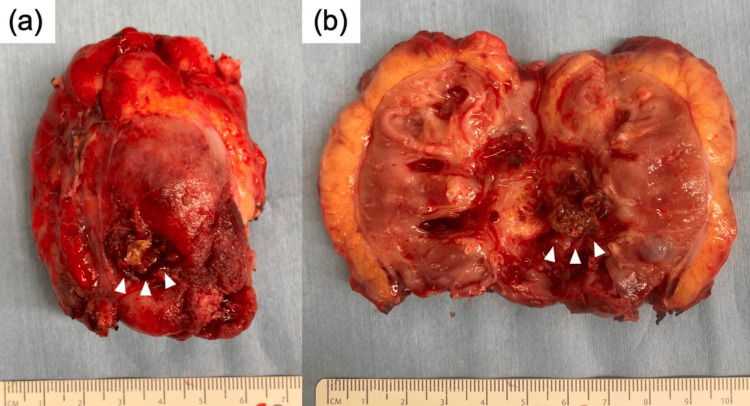
Findings of the renal specimen White arrowheads denote fistulas with infilled renal stones on (a) the dorsal side and (b) the pelvic side of the kidney.

The postoperative course was uneventful, and the patient was discharged on postoperative day 8.

Histopathological examination revealed marked neutrophilic inflammatory cell infiltration and abscess formation in the perirenal interstitium. The renal cortex was severely atrophic, with approximately 70% of glomeruli showing global sclerosis, accompanied by focal lymphocytic infiltration and hyaline arteriolosclerosis. No malignant findings were identified. Given the clinical findings of chronic infection, renal calculi, a nonfunctioning kidney, nephrocutaneous fistula formation, and severe adhesions, xanthogranulomatous pyelonephritis was strongly considered in the differential diagnosis. However, no histopathological findings suggestive of xanthogranulomatous pyelonephritis, such as xanthogranulomatous inflammation or foamy histiocyte infiltration, were observed, and the final pathological diagnosis was chronic suppurative pyelonephritis.

## Discussion

The main symptoms of NCF, such as back pain and fever, are often mild or absent: up to 63% of patients are asymptomatic at presentation, and only 30% present with flank pain [1-3]. Our patient initially presented with fever and flank pain, which resolved after spontaneous drainage of the abscess through a fistula. This suggests that the flank pain was caused by increased pressure from the perirenal abscess.

Although thick anatomical structures normally separate the kidney from the skin, certain regions, such as the lumbar triangle and lumbar quadrilateral, represent areas of low anatomical resistance through which fistulous tracts may extend to the flank skin [2,4]. In our patient, prolonged immobility due to bedridden status likely led to muscle atrophy and tissue fragility, while the dorsally located renal stone, close to the skin, may have facilitated fistula formation.

Contrast-enhanced CT plays a central role in NCF diagnosis, allowing both the identification of the fistulous tract and assessment of renal function, both of which are critical for treatment planning [2]. Fistulography is generally reserved for cases in which CT findings are inconclusive [2]. In our patient, CT clearly demonstrated continuity between the kidney and the skin, enabling a straightforward diagnosis. Furthermore, the affected kidney was confirmed to be nonfunctioning, and additional imaging was deemed unnecessary.

NCF typically results from long-standing inflammation, and approximately 90% of affected kidneys are nonfunctional at diagnosis [1]. In our patient, long-term ureteral stenting, persistent renal calculi, and recurrent pyelonephritis likely contributed to the chronic inflammation, progressive parenchymal thinning, and eventual loss of renal function. Nephrectomy is therefore considered the definitive treatment for most cases of NCF and is commonly performed using an open approach [1,2]. Although heminephrectomy has been reported in rare cases of preserved renal function [2], extensive inflammation and fibrosis generally preclude nephron-sparing surgeries.

Nephrectomy for NCF is often technically demanding because of the severe perinephric adhesions caused by chronic infection, which make anatomical orientation and dissection particularly challenging. Manohar et al. [5] demonstrated that laparoscopic nephrectomy for inflammatory renal conditions can be safely performed in selected patients with renal length < 10 cm without hilar lymphadenopathy or fibrosis around the renal artery. Patients who did not meet these criteria had a significantly higher risk of perioperative complications. Manohar et al. also noted that conversion to open surgery was more frequent on the right side than on the left, primarily because of the difficulty in identifying the dissection plane between the kidney and adjacent bowel [5]. Hsiao and Pattaras identified pyonephrosis as an important risk factor for conversion to open surgery [6]. In our patient, preoperative CT demonstrated marked perihilar fat stranding and significant hilar lymphadenopathy (Figures 3a, 3b), which were predictive of severe adhesions. These imaging findings correlated with intraoperative difficulties, particularly around the renal vein, ultimately necessitating partial resection of the IVC. Considering these factors, as well as the need for adequate debridement and irrigation of the infected field and the patient’s inability to tolerate positions other than supine, we opted for open surgery in our patient.

## Conclusions

NCF is a rare but clinically important complication in patients with renal stones and recurrent tract infections. In most cases, the affected kidney is nonfunctioning, and nephrectomy remains the treatment of choice. Preoperative CT findings such as marked perihilar fat stranding and hilar lymphadenopathy may suggest severe hilar adhesions and should alert surgeons to the possibility of technically difficult dissection and vascular injury.
